# Gender-specific pathways in mental health crisis in adolescents, from consultation to (in)voluntary admission: a retrospective study

**DOI:** 10.1186/s12888-024-05680-9

**Published:** 2024-03-28

**Authors:** Linda Dil, Saskia Mérelle, Nick Lommerse, Jaap Peen, Pety So, Rien Van, Jeroen Zoeteman, Jack Dekker

**Affiliations:** 1 NPI, Arkin, Buikslotermeerplein 420, 1025 WP Amsterdam, The Netherlands; 2 Department of Clinical Psychology, VU Faculties, De Boelelaan 1105, 1081 HV Amsterdam, The Netherlands; 3 Research Department 113 Zelfmoordpreventie, Paasheuvelweg 25, 1105 BP Amsterdam, The Netherlands; 4 Research Department Arkin, Klaprozenweg 111, 1033 NN Amsterdam, The Netherlands; 5 Youz, Center for Youth Mental Healthcare, Lupinestraat 1, 2906CV Capelle a/d IJssel, The Netherlands; 6 NPI, Arkin, Domselaerstraat 126, 1093 MB Amsterdam, The Netherlands; 7 Psychiatric Emergency Service, Arkin, 1 e Constantijn Huijgensstraat 38, 1054 BR Amsterdam, The Netherlands

**Keywords:** Adolescents, Crisis, Predictors of hospitalisation, Emergency psychiatry, Gender differences

## Abstract

**Background:**

A strong increase in mental health emergency consultations and admissions in youths has been reported in recent years. Although empirical evidence is lacking, gender differences in risk of admission may have contributed to this increase. A clearer understanding of the relationship, if any, between gender and various aspects of (in)voluntary care would help in more evidence-based service planning.

**Methods:**

We analysed registry data for 2008–2017 on 3770 outpatient emergencies involving young people aged 12 to 18 years from one urban area in the Netherlands, served by outreaching psychiatric emergency services. These adolescents were seen in multiple locations and received a psychosocial assessment including a questionnaire on the severity of their problems and living conditions. Our aims were to (a) investigate the different locations, previous use of mental health service, DSM classifications, severity items, living conditions and family characteristics involved and (b) identify which of these characteristics in particular contribute to an increased risk of admission.

**Results:**

In 3770 consultations (concerning 2670 individuals), more girls (58%) were seen than boys. Boys and girls presented mainly with relationship problems, followed by disruptive disorders and internalizing disorders. Diagnostic differences diminished in hospitalisation. More specifically, disruptive disorders were evenly distributed. Suicide risk was rated significantly higher in girls, danger to others significantly higher in boys. More girls than boys had recently been in mental health care prior to admission. Although boys and girls overall did not differ in the severity of their problems, female gender predicted admission more strongly. In both boys and girls severity of problems and lack of involvement of the family significantly predicted admission. Older age and danger to others significantly predicted admission among boys, whereas psychosis, suicidality and poor motivation for treatment predicted admission among girls.

**Conclusion:**

There are different pathways for youth admission, which can partly be explained by different psychiatric classifications as well as gender-specific differences with regard to age, suicide risk, danger to others and the influence of motivation for treatment. Finally, for both genders, family desire for hospitalisation is also an important predictor.

## Background

Mental health crises have led to a sharp increase in both consultations by psychiatric emergency services (PES) and hospital admissions in youths over the past years [1–3]. Uncertainties remain regarding different pathways to emergency mental health care for boys and girls, especially regarding the factors leading to (in)voluntary admission [4]. Different legal criteria for involuntary hospitalisation and differences in mental health systems and socio-economic status have influenced the knowledge base so far, sometimes hindering generalisability [5]. More knowledge about gender-specific characteristics that may lead to an increased risk of admission could help to ensure that mental health services meet the needs of boys and girls equally.

### Gender-specific characteristics of adolescent mental health

Gender differences in mental health tend to emerge in later childhood and adolescence. Adolescent girls have a substantially higher prevalence of depression and eating disorders as well as suicidal ideation and suicide attempts than boys. Adolescent boys are more likely than girls to exhibit behavioural problems and death by suicide [6]. In addition, girls are affected by lower self-esteem, a greater propensity for body shame and rumination, and higher rates of childhood sexual abuse [7]. During adolescence, in addition to biological changes, gender norms become ingrained, shaping mental health and help-seeking. Stereotypically, boys may learn to hide their vulnerabilities and health needs by refusing to seek care. Gender norms can make it easier for girls to express their feelings and seek help [8]. Moreover, the environment can have different effects on the expression of psychopathology in boys and girls, providing risk or protection [9].

### Gender characteristics from consultation to (in)voluntary admission

In the routing from crisis consultation to inpatient admission, both differences and similarities were found between boys and girls. Boys with behavioural problems are predominantly seen in the younger group of children (4–12 years) [10–12]. In the 12–18 age group, usually more girls are seen because of internalizing disorders [1, 13]. Girls returned more often after an initial consultation [14], were seen more often in general hospitals after autointoxication [15] and showed more comorbidities and a higher risk of suicide or self-harm when autism was present [16]. For both genders, social/interpersonal problems, parental mental illness, increasing rates of comorbidity, family disruption and family demands for consultation were important environmental factors [2, 3, 11, 17]. Girls are usually admitted more often due to self-harm, suicidality and eating disorders [13, 18, 19]. Hospitalised boys show more behavioural disorders, psychosis and substance abuse.

It can be concluded that there is evidence of relevant differences between boys and girls in crisis. This is one of the first studies to examine predictors of admission by gender and then compare them within a considerably large representative sample of adolescents.

The aims of this study were to:

1. Examine the type, severity and circumstances in consultations with adolescents aged 12 to 18 leading to an increased risk of admission.
2. Investigate whether these characteristics differ between boys and girls.

We expect girls to be assessed more often in general hospitals and subsequently admitted more often than boys because of internalizing problems and suicide risk/self-harm. In contrast, boys are expected to be assessed more often at a police station and then admitted mainly because of substance abuse, psychosis and externalizing disorders. We expect that for both boys and girls, the severity of the clinical situation and related circumstances contribute equally to the risk of admission.

## Methods

### Setting

The study was conducted in the urban area of Amsterdam in the Netherlands (approximate population 1.4 million). In this region, an outreaching psychiatric emergency service (PES) is responsible 24/7 for assessing all patients referred to it. In Amsterdam, the PES consists of a specialised outreach team for children and adolescents, which provides an after-hours service for both childcare/custody protection services and regular mental health providers. During office hours, regular mental healthcare providers and childcare/custody protection services have their own outreaching teams to cater to emergency patients. Most patients are referred by telephone by general practitioners, mental health care workers, police, and the emergency departments (EDs) of general hospitals. When police officers are confronted with problematic behaviour, they call a mental health nurse from the City Health Service, who performs the psychiatric triage, before calling in the PES.

The primary tasks of the outreach PES are triage, assessment and subsequent referral of psychiatric emergency patients to other psychiatric services. The staff at the emergency services consists of community psychiatric nurses, physicians, psychologists and psychiatrists who are all trained to make psychiatric diagnoses and emergency interventions. They determine whether an acute assessment is necessary based on information obtained over the phone. If so, patients of all ages are examined at the patient’s location by a team consisting of a psychologist or nurse and a physician or psychiatrist. If the physician is not a psychiatrist, a psychiatrist is consulted by telephone. If necessary, a child psychiatrist in the emergency ward of a general hospital can be consulted. Since the police in the Netherlands are not allowed to take mentally ill children to a psychiatric hospital, they usually ask for psychiatric emergency-service staff to examine them at the police station. In all situations, at a police station or elsewhere, the PES assesses the patient and, when applicable, their significant others and attempts to resolve the crisis, preferably without hospitalisation. In the Netherlands, the conditions for forced hospitalisation are having a mental disorder, the presence of danger to self-and/or others, lack of alternatives to avert the danger, in conjunction with resistance to hospitalisation.

### Patients

Registry data (2008–2017) were analysed from 3770 outpatient emergencies involving children aged 12–18 years referred to mobile PES in one urban area. We included all emergencies of youths who had been referred for urgent consultation.

### Data extraction

Well-trained professionals collected demographic and contextual factors, as well as clinical characteristics. Data were processed in accordance with the General Data Protection Regulation.

Demographic characteristics included age, gender, and living situation (two-parent family, single-parent family, mental health facility, vagrancy/homelessness, ‘other’ and unknown). Specific data on socioeconomic status and educational attainment levels were not recorded. Contextual characteristics included place of consultation, previous use of mental health service and ‘does the family want admission?’ (yes, no or unknown).

Clinical characteristics consisted of DSM-IV classifications [20], recent drug or alcohol use (yes/no/unknown), and severity of problems. The DSM-IV Axis I classifications were grouped into six categories: Psychotic spectrum disorders, Internalizing Disorders (including Depressive and Anxiety disorders and PTSD), Externalizing Disorders (Conduct disorders, PDD-NOS, attention-deficit disorders, impulse-control disorders), drug abuse, relationship problems (parent-child relational problems and problems related to abuse or neglect), and ‘other’. A disorder was recorded as present if it was classified on Axis I; more than one diagnostic category could be recorded for an individual patient. Axis II classification was not systematically recorded; therefore, this variable was not included in our analysis.

The severity of specific problems was assessed using the Severity of Psychiatric Illness scale (SPI) [21, 22]. The SPI is a decision-support tool to assess the need for services. It provides a structured description of the severity of psychopathology and of possible complications regarding the disorder and regarding treatment. As the SPI was only validated for use with adults, each PES professional was trained to apply the questionnaire with a selection of age-appropriate (7 out of 14) SPI items: suicide risk, danger to others, severity of symptoms, family disruption, residential instability, lack of motivation for treatment, lack of family involvement, and we added family’s wish for admission. These items were scored on a four-point scale from 0 (no problem) to 3 (severe problem).

The outcome of the emergency consultations was also available. Intervention was defined as voluntary admission, compulsory admission, and no admission.

### Analysis

Descriptive statistics were used to calculate the characteristics of the psychiatric emergency consultations for boys and girls separately. Pearson’s chi-square tests and independent t tests were then used to test group differences. *P* values of ≤ 0.05 were considered statistically significant. Standardised residuals (SR) were then analysed to measure which categories contributed most to the significant chi-square value. For this purpose, the four categories of the SPI items ‘suicide risk’ and ‘danger to others’ were dichotomised into the categories low/none and medium/high, while the SPI item ‘severity of disorder’ was changed into 3 categories with ‘low and moderate’ combined. Stepwise logistic regression analyses were used to identify factors that predict (voluntary or compulsory) admission to a psychiatric hospital, stratified by gender to account for possible gender differences. The regression analyses included all individual categories of the aforementioned variables. However, the categories psychiatric services, general hospital and other were combined for the variable location of consultation, and the categories psychotic disorder and substance use disorder were combined for the variable diagnosis. Finally, the goodness of fit of the models was assessed using Cox and Snell to calculate their explained variance. SPSS version 27 was used for all statistical analyses.

## Results

### Consultations

A total of 3770 consultations were conducted, in most cases (53.2%), at the police station, followed by home assessment (16.9%), mental health care institution (13.0%) and general hospital (3.3%). More girls (57.9%) than boys (42.1%) were seen. A total of 2670 adolescents (1123 boys and 1547 girls) were involved, a quarter of whom required multiple consultations, with gender being equally distributed. Table 1 shows information on the location of assessment, classification set, some SPI items, living conditions and family involvement.

**Table 1:** Characteristics of emergency consultations stratified by gender

Characteristic	Consultations Boys (12–18 year)	Consultations Girls	Overall Sample	T-/χ^2^	*P*-value
	*N* = 1583	*N* = 2187	*N* = 3770		
Age in years, mean (SD)	15.6 (1.6)	15.5 (1.5)	15.5 (1.6)	2.743	0.293
Living situation, n (%)				10.266	0.068
With single parent	380 (24.0)	515 (23.5)	895 (23.7)		
With 2 parents	287(18.1)	434 (19.8)	721 (19.1)		
Mental health facility	19(1.2)	16 (0.7)	35 0.9)		
Vagrant, homeless	30 (1.9)	21 (1.0)	51 (1.4)		
Other	87 (5.5)	134 (6.1)	221 (19.1)		
Unknown	780 (49.3)	1067 (48.8)	1847 (49.0)		
DSM-IV Axis 1 disorder, n (%)				99.626	*P* < 0.001
Relationship problems *	840 (53.1)	1312 (60.0)	2152 (57.1)		
Externalizing disorder **	402 (25.4)	367 (16.8)	769 (20.4)		
Internalizing disorder **	129 (8.1)	272 (12.4)	401 (10.6)		
Psychotic disorder **	65 (4.1)	36 (1.6)	101 (2.7)		
Substance use disorder **	53 (3.3)	29(1.3)	82 (2.2)		
Other Axis 1 disorder *	94 (5.9)	171 (7.8)	265 (7.0)		
Location, n (%)				28.412	*P* < 0.001
Police station	859 (54.3)	1145 (52.4)	2004 (53.2)		
At home	292 (18.4)	344 (15.7)	636 (16.9)		
Regular Outpatient Clinic	209 (13.2)	281 (12.8)	490 (13.0)		
General hospital **	28 (1.8)	98 (4.5)	126 (3.3)		
Other	195 (12.3)	319 (14.6)	514 (13.6)		
Previous service use, n (%)				15.042	*P* < 0.01
Less than 3 months prior	1013 (64.0)	1345 (61.5)	2358 (62.5)		
Longer than 3 months prior	68 (4.3)	104 (4.8)	172 (4.6)		
None *	373 (23.6)	610 (27.9)	983 (26.1)		
Unknown *	129 (8.1)	128 (5.9)	257 (6.8)		
Suicide risk, n (%)				28.591	*P* < 0.001
Medium to high risk	80 (5.1)	214 (9.8)	294 (7.8)		
Low to none	1503 (94.9)	1973 (90.2)	3476 (92.2)		
Danger to others, n (%)				124.394	*P* < 0.001
Likely, very likely	367 (23.2)	216 (9.9)	583 (15.5)		
No	1216 (76.8)	1971 (90.1)	3187 (84.5)		
Severity of symptoms, n (%)				6.237	0.044
Severe	108 (6.8)	150 (6.9)	258(6.8)		
Low– Moderate*	551 (34.8)	678 (31.0)	1229 (32.6)		
None	924 (58.4)	1359 (62.1)	2283 (60.6)		
Motivation for treatment, n (%)				8.048	0.045
High	1004 (63.4)	1466 (67.0)	2470 (65.5)		
Medium	295 (18.6)	393 (18.0)	688 (18.2)		
Low*	194 (12.3)	211 (9.6)	405 (10.7)		
None	90 (63.4)	117 (5.3)	207 (5.5)		
Residential instability, n (%)				7.790	0.051
Severe *	155 (9.8)	163 (7.5)	318 (8.4)		
Moderate	177 (11.2)	486 (22.2)	423 (11.2)		
Low	309 (19.5)	554 (25.3)	717 (19.0)		
None	942 (59.5)	804 (36.8)	2312 (61.3)		
Family disruption, n (%)				3.528	0.317
Severe	267 (16.9)	343 (15.7)	610 (16.2)		
Moderate	315 (19.9)	486 (22.2)	801 (21.2)		
Low	399 (25.2)	554 (25.3)	953 (25.3)		
None	602 (38.0)	804 (6.8)	1406 (37.3)		
Family involvement, n (%)				4.114	0.249
High	1210 (76.4)	1671 (76.4)	2881 (76.4)		
Medium	227 (14.3)	336 (15.4)	563 (14.9)		
Low	108 (6.8)	146 (6.7)	254 (6.7)		
None	38 (2.4)	34 (1.6)	72 (1.9)		
Family wanted admission, n (%)				5.683	0.058
Yes* No Not applicable	159 (10.0) 124 (7.8) 1300 (82.1)	176 (8.0) 197 (9.0) 1814 (82.9)	335 (8.9) 321 (8.5) 3114 (82.6)		

* Standardised residuals > 1.5 ** Standardised residuals > 2.7

Regarding the location of the assessment, significantly more girls (4.5%, SR > 2.7) were assessed in a general hospital than boys (1.8%). There were no gender differences regarding the other locations. It is, however, notable that the group of girls (52,4%) assessed at a police station was almost as large as the group of boys (54.3%). About two-thirds of the youngsters were in mental health care, with gender being equally distributed. Regarding the clinical ratings set, there were several significant differences. Among boys, 3.3% were rated as substance use disorder, 4.1% as psychotic disorder and 25.4% as externalizing disorder compared with 1.3%, 1.6% and 16.8% among girls, respectively. In contrast, relationship problems and depressive and anxiety disorders were found significantly more often in girls. There was a difference between boys and girls in terms of severity of the condition, in the low-moderate range with boys rating 34.8%, SR > 1.5 and girls 31%. Suicide risk in girls (9.8%) was rated two times higher than in boys (5.1%, *p* < 0.001). In contrast, high danger to others was rated twice as often in boys compared to girls (23.2% and 9.9%, respectively, *p* < 0.001). With regard to living situation boys were more often faced with a severely unstable living situation (9.8%, >SR 1.5).

### Admissions

After assessment, hospital admission followed in 194 cases (5.1% of 3770 consultations). These included 77 boys (4.9% of total consultations in boys) and 117 girls (5.3% of total consultations in girls, *p* = 0.50). The largest group of adolescents came through mental health institutions (52,6%). Assessment at the general hospital was significantly more common among girls (16,2%, SR > 1.5). Significantly more girls (80,3%, SR > 1.5) than boys (53.2%) had been in mental health care less than three months previously, while more boys (19,5%, SR > 1.5) had not been in mental health care at all. Within the admitted adolescent group, it is notable that the differences in classifications between boys and girls narrowed. Only psychotic disorders and substance use were significantly more common in boys, and mood disorders were more common in girls (29,9%, SR > 2.7). Once admitted, the group with externalizing disorders was equal among boys and girls. Regarding living situation and other family characteristics, no significant differences were found, including the family’s desire to have their child admitted.

Legal status at admission was evenly distributed (*p* = 0.876) (Table 2). Again, suicidality in girls was more often rated as ‘moderate to high’ (*p* < 0.01), whereas boys more often showed danger towards others (*p* < 0.001). Boys and girls were equally motivated for treatment.

**Table 2:** Clinical characteristics of youths admitted to psychiatric hospitals after emergency consultations stratified by gender

Characteristic	Boys	Girls	Overall Sample	T-/χ^2^	*P*-value
	*N* = 77	*N* = 117	*N* = 194		
Age in years, mean (SD)	16.40 (1.50)	16.24 (1.34)	16.30 (1.40)	1.151	0.934
Living situation, n (%)				5.440	0.365
With single parent	17 (22.1)	19 (16.2)	36 (18.6)		
With 2 parents	16 (20.8)	40 (34.2)	56 (28.9)		
Mental health facility	6 (7.8)	6 (5.1)	12 (6.2)		
Vagrant, homeless	2 (2.6)	1 (0.9)	3 (1.5)		
Other	6 (7.8)	7 (6.0)	13 (6.7)		
Unknown	30 (39.0)	44 (37.6)	74 (38.1)		
DSM-IV Axis 1 disorder, n (%)				26.781	*P* < 0.001
Relationship problems	9 (11.7)	10 (8.5)	19 (9.8)		
Externalizing disorder	16 (20.8)	40 (34.2)	56 (28.9)		
Internalizing disorder **	11 (14.3)	35 (29.9)	46 (23.7)		
Psychotic disorder **	34 (44.2)	20 (17.1)	54 (27.8)		
Substance use disorder *	6 (7.8)	3 (2.6)	9 (4.6)		
Other Axis 1 disorder	1 (1.3)	9 (7.7)	10 (5.2)		
Location				9.378	0.052
Police station	9 (11.7)	10 (8.5)	19 (9.8)		
At home	11 (14.3)	15 (12.8)	26 (13.4)		
Regular Outpatient Clinic	45 (58.4)	57 (48.7)	102 (52.6)		
General hospital *	2 (2.6)	19 (16.2)	21 (10.8)		
Other	10 (13.0)	16 (13.7)	26 (13.4)		
Previous service use, n (%)				16.947	*P* < 0.01
Less than 3 months prior *	41 (53.2)	94 (80.3)	120 (61.9)		
Longer than 3 months prior	2 (2.6)	2 (1.7)	4 (2.1)		
None *	15 (19.5)	7 (6.0)	22 (11.3)		
Unknown *	19 (24.7)	14 (12.0)	33 (17.0)		
Type of admission, n (%)				0.099	0.167
Voluntary	24 (31.2)	39 (33.3)	63 (32.5)		
Compulsory	53 (68.8)	78 (66.7)	131 (67.5)		
Suicide risk, n (%)				8.118	*P* < 0.01
Medium to high risk	28 (36.4)	67 (57.3)	95 (49.0)		
Low to none	49 (63.6)	50 (42.7)	99 (51.0)		
Danger to others, n (%)				44.619	*P* < 0.001
Likely, very likely	73 (94.8)	57 (48.7)	130 (67.0)		
No	4 (5.2)	60 (51.3)	64 (51.3)		
Severity of symptoms, n (%)				3.581	0.167
Severe	52 (67.5)	79 (67.5)	131 (67.5)		
Low– Moderate	24 (31.2)	30 (25.6)	54 (27.8)		
None	1 (1.3)	8 (6.8)	9 (4.6)		
Motivation for treatment, n (%)				0.365	0.947
High	6 (7.8)	12 (10.3)	18 (9.3)		
Medium	19 (24.7)	27 (23.1)	46 (23.7)		
Low	21 (27.3)	31 (26.5)	52 (26.8)		
None	31 (40.3)	47 (40.2)	78 (40.2)		
Residential instability, n (%)				5.204	0.157
Severe *	23 (29.9)	20 (17.1)	43 (22.2)		
Moderate	14 (18.2)	21 (17.9)	35 (18.0)		
Low	9 (11.7)	13 (11.1)	22 (11.3)		
None	31 (40.3)	63 (53.8)	94 (48.5)		
Family disruption, n (%)				5.833	0.120
Severe	26 (33.8)	42 (35.9)	68 (35.1)		
Moderate	24 (31.2)	26 (22.2)	50 (25.8)		
Low	18 (23.4)	21 (17.9)	39 (20.1)		
None	9 (11.7)	28 (23.9)	37 (19.1)		
Family involvement, n (%)				0.105	0.991
High	48 (62.3)	72 (61.5)	120 (61.9)		
Medium	14 (18.2)	23 (19.7)	37 (19.1)		
Low	10 (13.0)	14 (12.0)	24 (12.4)		
None	5 (6.5)	8 (6.8)	13 (6.7)		
Family wanted admission, n (%)				1.818	0.403
Yes	56 (72.7)	79 (67.5)	135 (69.6)		
No	2 (2.6)	8 (6.8)	10 (5.2)		
Not applicable	19 (24.7)	30 (25.6)	49 (25.3)		

* Standardised residuals > 1.5 ** Standardised residuals > 2.7

### Determinants of hospitalisation by gender

A comprehensive multiple logistic regression model with consultations of boys and girls together, containing all the variables of Tables 3 and 4 with the addition of gender, showed that male gender predicted admission less strongly than female gender (OR = 0.45, 95% CI range = 0.27–0.76, *p* = 0.003). Following is the discussion of the final models, as shown in Tables 3 and 4, which break down different predictors by gender. Older age predicted admission in boys. Neither specific location, nor previous use of mental health services significantly increased the likelihood of hospitalisation. In particular severity of problems (OR = 3.33, 95% CI = 1.95–5.67), followed by danger to others (OR = 4.83, 95% CI = 2.78–8.39), lack of family involvement (OR = 1.89, 95% CI = 1.11–3.24) and the family’s desire for hospitalisation of the child (OR = 15.27, 95%-CI = 2.92–79.94) were strong predictors.

**Table 3:** Clinical characteristics predicting admission to psychiatric hospital among boys resulting from stepwise logistic regression analysis

Admission to psychiatric hospital among boys– yes (*N* = 77) or no (*N* = 1583 consultations)									
Characteristic	First model			Second model			Final model		
	OR	95% CI	*P*	OR	95% CI	*P*	OR	95% CI	*P*
Age	1.133	0.93–1.38	0.209	1.384	1.05–1.82	0.020	1.380	1.02–1.86	0.034
DSM-IV Axis 1 disorder (ref: internalizing disorder)									
Relationship problems	0.225	0.09–0.58	0.002	0.909	0.24–3.47	0.889	1.557	0.33–7.34	0.576
Externalizing disorder	0.718	0.31–1.66	0.438	0.536	0.16–1.76	0.303	0.676	0.18–2.59	0.567
Psychotic disorder/substance use disorder	4.073	1.87–8.87	0.000	1.115	0.37–3.38	0.847	1.135	0.32–4.04	0.845
Other Axis 1 disorder	0.166	0.02–1.36	0.095	0.281	0.02–3.39	0.318	0.687	0.05–8.70	0.772
Location of consultation (ref: at home)									
Police station	0.332	0.15–0.76	0.009	0.928	0.32–2.68	0.890	1.415	0.44–4.58	0.562
Other	2.740	1.49–5.04	0.001	1.753	0.79–3.91	0.170	1.575	0.64–3.87	0.322
Previous service use (ref: < 3 months)									
> 3 months	1.328	0.29–6.17	0.717	3.977	0.32–49.62	0.284	2.065	0.14–31.02	0.600
None	1.004	0.20–5.01	0.996	17.515	0.24–44.25	0.374	2.671	0.17–42.08	0.108
SPI Items (ref: No)									
Suicide risk				1.429	0.92–2.21	0.110	1.558	0.99–2.47	0.058
Danger to others				4.632	2.81–7.64	0.000	4.829	2.78–8.39	0.000
Severity of symptoms				3.483	2.14–5.66	0.000	3.327	1.95–5.67	0.000
Lack of motivation for treatment				1.067	0.71–1.61	0.754	0.853	0.54–1.34	0.490
Residential instability				1.347	0.99–1.84	0.060	1.162	0.77–1.77	0.481
Family disruption							0.950	0.60–1.51	0.830
Lack of family involvement							1.894	1.11–3.24	0.019
Family wanted admission (ref: No)									
Yes							15.271	2.92–79.94	0.001
Not applicable							1.731	0.31–9.68	0.532
Cox and Snell R^2^	0.12			0.21			0.23		

**Table 4:** Clinical characteristics predicting admission to psychiatric hospital among girls resulting from stepwise logistic regression analysis

Admission to psychiatric hospital among girls– yes (*N* = 177) or no (*N* = 2187 consultations)									
Characteristic	First model			Second model			Final model		
	OR	95% CI	*P*	OR	95% CI	*P*	OR	95% CI	*P*
Age	1.102	0.95–1.29	0.215	0.947	0.79–1.14	0.565	1.069	0.87–1.32	0.532
DSM-IV Axis 1 disorder (ref: internalizing disorder)									
Relationship problems	0.164	0.08–0.35	0.000	0.720	0.30–1.73	0.462	0.686	0.27–1.78	0.437
Externalizing disorder	1.418	0.83–2.43	0.204	2.339	1.20–4.58	0.013	2.136	0.99–4.60	0.052
Psychotic disorder/substance use disorder	4.090	2.01–8.34	0.000	4.357	1.69–11.22	0.002	4.726	1.64–13.62	0.004
Other Axis 1 disorder	0.755	0.34–1.69	0.494	1.159	0.42–3.20	0.776	0.726	0.23–2.27	0.582
Location of consultation (ref: At home)									
Police station	0.252	0.12–0.53	0.252	0.737	0.31–1.77	0.496	0.678	0.26–1.74	0.418
Other	3.580	2.21–5.80	3.580	1.710	0.96–3.04	0.068	1.520	0.81–2.87	0.197
Previous service use (ref: <3 months									
> 3 months	3.768	0.85–16.70	0.081	1.368	0.28–6.61	0.697	1.285	0.23–7.28	0.777
None	0.657	0.13–3.455	0.62	0.402	0.07–2.35	0.311	0.546	0.08–3.86	0.545
SPI Items (ref: No)									
Suicide risk				1.878	1.39–2.54	0.000	1.619	1.15–2.27	0.005
Danger to others				1.545	1.08–2.22	0.018	1.390	0.93–2.09	0.111
Severity of symptoms				2.613	1.80–3.80	0.000	2.269	1.49–3.47	0.000
Lack of motivation for treatment				1.809	1.38–2.37	0.000	1.518	1.13–2.05	0.006
Residential instability				1.185	0.93–1.51	0.175	1.178	0.85–1.63	0.320
Family disruption							0.789	0.57–1.10	0.158
Lack of family involvement							1.557	1.06–2.30	0.025
Family wanted admission (ref: No)									
Yes							15.709	6.15–40.14	0.000
Not applicable							1.650	0.62–4.41	0.318
Cox and Snell R^2^	0.13			0.20			0.22		

In girls, age did not predict admission. Again, neither specific location, nor previous service use increased the likelihood of hospitalisation. In particular psychosis (OR = 4.73, 95%-CI = 1.64–13.62) predicted admission. Four SPI items showed significant predictive value, with suicide risk (OR = 1.62, 95%-CI = 1.15–2.27) and severity of disorder (OR = 2.27, 95%-CI = 1.49–3.47) weighing most heavily, followed by lack of motivation and poor family involvement. Family’s desire for hospitalisation was the predictor with the highest odds ratio (OR = 15.71, 95%-CI = 6.15–40.14).

## Discussion

### Main findings

This study finds some striking differences but also similarities between boys and girls in the route to inpatient admission. Not only do girls come for consultation more often, female gender also predicts admission significantly more strongly than male gender, while the high severity of the condition is initially estimated to be the same for boys and girls. At the police station, the main finding place for both, approximately equal numbers of boys and girls are seen, mainly because of relationship problems. In boys, significantly more psychosis, substance abuse and externalizing disorders are subsequently seen with danger to others. In girls, more internalizing problems with a higher estimated risk of suicide are seen. In the admitted situation, no differences are found with regard to legal status, family circumstances, motivation for treatment, and the differences in classifications decrease. Significantly more girls than boys had been in mental health care less than three months prior to the admission, and more boys than girls had not been in mental health care at all. In boys, the final risk of admission is mainly increased by older age and danger to others. In both genders, severity of the problems and lack of involvement of the family predicted admission, but previous use of mental health service and location of consultation did not. Unlike boys, girls were more likely to be admitted when they were psychotic, suicidal or were poorly motivated for treatment. In particular, the family’s desire to have the child hospitalised was an important predictor in both boys and girls.

The fact that girls are seen for crisis consultation more often than boys may partly be because they seek help earlier [23]. In addition, a remarkable number of girls are seen for consultation at a police station. If the crisis service is used as a thermometer of care, which can show societal trends [24], this could show that girls are catching up in terms of externalizing disorders. In fact, in the hospitalised group, it was the most common disorder among girls. Earlier findings in the literature that, compared to boys, girls are mostly seen in crisis because of internalizing disorders are thus nuanced. Girls seem to seek help for both internalizing and increasingly externalizing problem behaviours.

The police station was the main finding place (53%) for both genders and did not lead to a higher risk of hospitalisation. In comparison, it was found in the state of Florida, U.S., that police officers initiated 67% of involuntary psychiatric holds of minors [25]. In Europe the rate of police involvement is expected to be lower [26], but unfortunately, the literature on this aspect of crisis intervention in minors is very scarce.

Not only differences in crisis intervention were found between countries, but also between regions in the Netherlands. In the Amsterdam region a high proportion of police involvement (44%) in emergency presentations in adults was also found, which was related to the high population density and high prevalence of severe pathology in this region [27]. Differences in the organisation of acute care for minors by region make it difficult to draw similar comparisons. When we initially analysed the youth data from both Amsterdam and Rotterdam, we found large differences in outcomes by region. This high variance between regions in the characteristics of youth seeking emergency care, called for an understanding. It became clear that data on outreaching crisis care by the Youth Welfare system in the Rotterdam catchment area were lacking. This finding motivated us post-hoc to apply our analyses exclusively to the Amsterdam region, the region with the highest number of consultations, generating representative outcomes for this region.

In the Netherlands, the most common alternative pathway for psychiatric hospitalisation of minors is containment by the Youth Welfare system, followed by placement with extended family or uptake by the judicial system [11], besides ambulatory care. With only 5,1% of the consultations leading to psychiatric hospitalisation in this sample, it remains important for psychiatric services to join forces with the Youth Welfare and judicial system in order to serve these youngsters, as it is well known that psychiatric problems are also common and equally severe in the latter groups [28].

A total of 53% were admitted through an (outpatient) clinic, where more girls than boys had been in mental health care less than three months previously and more boys than girls had not been in care at all. Six out of ten admissions were girls. In comparison, in the South of the Netherlands, it was found that eight out of ten admissions on a youth crisis ward were girls [29]. Taken together, these findings may indicate that there is a need to lower the threshold for mental health care for boys with severe mental health problems.

Overall, suicidality was significantly more often estimated as severe in girls and in contrast to boys, suicidality was a significant predictor of hospital admission. Given that boys are two to three times more likely than girls to die by suicide [30], it is possible that boys express themselves less clearly in this regard [31] or that a cry for help is not recognised in a timely manner due to a focus on externalizing problem behaviour, which in some cases is followed by criminal justice treatment. Indeed, in this study, danger to others was rated approximately twice as high in boys. Hawton et al. [32] nevertheless argued that predicting suicide is very difficult and can lead to defensive action by care providers. They advocated a shift from predicting to a more therapeutic approach as early as the time of crisis assessment. By extension, crisis services could also serve as an opportunity for the indicated prevention of adolescents at increased risk of personality disorders. Since an important group of both boys and girls present first and foremost with relationship problems in crisis situations, this approach might seamlessly fit identified cases.

Female gender was more predictive of admission, even when high severity of the problems is rated evenly high in both genders. When girls were psychotic, suicidal or were poorly motivated, these circumstances were significant predictors of admission; in boys, they were not. Does this perhaps mean that girls in such circumstances are more likely than boys to call for protection when they are in crisis? And that their autonomy is more likely to be taken over even if they are not motivated for treatment? Unfortunately, this situation sometimes leads to an increase in (para)suicidal behaviour in a subgroup of female adolescents [33]. This adverse turn of events highlights the importance of formulating possible gender-specific intervention strategies for PES.

Older age predicted admission in adolescent boys but not in girls. This coincides with the finding that girls pose less of a danger to others, perhaps safeguarding the holding capacity of the environment longer. Boys typically have difficulty regulating aggression in early and middle adolescence, which takes its toll in relationships with peers and family [34]. Girls may be protected from derailment relatively longer by stronger relational capacities and a pull toward dependency due to societal norms.

Finally, we found that the family’s desire for hospitalisation of the child was the strongest predictor of hospitalisation. Only in approximately 5% of the admitted boys and girls was the family opposed to hospitalisation. Therefore, regardless of psychiatric disorder and other severity factors, hospitalisation seems most imminent when the family’s capacity to contain is exhausted.

### Clinical implications

Gender-specific differentiated strategies in adolescents in crisis should be considered. In boys, the possibility of masked suicidality should be considered, even if predominantly externalizing problems are apparent. There is also a need to lower the threshold for mental health care for boys, both in the run-up to a serious crisis and when considering admission. In girls, on the other hand, sometimes hospitalisation needs to be prevented. Additional conditions such as psychosis, suicide risk and lack of motivation should equally prompt hospital admission in boys and girls. For both genders, further assessment of personality problems should be considered to promote the indicated prevention of personality disorder. Special focus is needed on helping families and support systems contain adolescents in crisis.

### Strengths, limitations and future directions

This study focuses on an important issue (the relationship between gender and specific aspects of hospital admission in adolescents) and comprises a considerably large sample size, which allowed a broad range of variables to be studied. The catchment area contains a diverse urban population of approximately 1.4 million people, promoting generalizability in a context of increasing urbanisation. We focused on patients referred to the PES. Other services, such as regular outpatient services for mental health or social and Youth Welfare services, should be taken into account when expressing an opinion on the total population of children and adolescents with urgent needs. Additionally, we used the SPI, a standardised decision-support tool. However, the SPI has been validated for adults, not for young people. We also considered whether the adolescent was already in care, as this circumstance is known to reduce the likelihood of admission [13]. Limitations are as follows: We did not consider the influence of comorbidity, although this has proven to be a factor that increases the risk of suicide [35]. Second, with regard to family circumstances, we considered family composition but not parental mental health, although the latter factor is particularly important for a child’s resilience [36]. Third, the DSM classifications were based on clinical interviews rather than standard formats for assessment. Fourth, we lacked information about Youth Welfare involvement and admission to youth facilities. Fifth, specific data on socioeconomic status and educational attainment levels were not recorded. Recommendations for future studies include exploring the validity and interrater reliability of the SPI (or other decision-support tool) in adolescents and exploring a possible gender bias among care providers using the tool. Second, future longitudinal studies should identify further patterns in the presentations of boys and girls that may influence outcomes for different psychiatric disorders, informing clinical decision making in the future.

## Conclusion

Adolescent boys and girls in a mental health crisis show different pathways to hospital admission. This can partly be explained by different psychiatric classifications as well as gender-specific differences with regard to age, suicide risk, danger to others and the influence of family circumstances and motivation. Enhanced gender sensitivity among care providers may thus improve decision-making regarding the need for admission. Finally, the family’s desire for admission was also a significant predictor of admission for both genders, indicating the unchanged importance of helping support systems to contain adolescents in crisis.

## Data Availability

The datasets used and/or analysed during the current study are available from the corresponding author on reasonable request.
